# Clinical utility of genomic sequencing: a measurement toolkit

**DOI:** 10.1038/s41525-020-00164-7

**Published:** 2020-12-15

**Authors:** Robin Z. Hayeems, David Dimmock, David Bick, John W. Belmont, Robert C. Green, Brendan Lanpher, Vaidehi Jobanputra, Roberto Mendoza, Shashi Kulkarni, Megan E. Grove, Stacie L. Taylor, Euan Ashley, Medical Genome Initiative

**Affiliations:** 1 Program in Child Health Evaluative Sciences, The Hospital for Sick Children and the Institute of Health Policy Management and Evaluation, University of Toronto, Toronto, ON Canada; 2 Rady Children’s Hospital Institute for Genomic Medicine, San Diego, CA USA; 3 HudsonAlpha Institute for Biotechnology, Huntsville, AL USA; 4 Illumina Inc., San Diego, CA USA; 5 Brigham and Women’s Hospital Broad Institute and Harvard Medical School, Boston, MA USA; 6 Mayo Clinic, Rochester, MN USA; 7 New York Genome Center, New York, NY USA; 8 Department of Pathology and Cell Biology Columbia University Medical Center, New York, NY USA; 9 The Division of Clinical and Metabolic Genetics, The Hospital for Sick Children, Toronto, ON Canada; 10 Baylor Genetics and Baylor College of Medicine, Houston, TX USA; 11 Department of Molecular and Human Genetics, Baylor College of Medicine, Houston, TX USA; 12 Stanford Medicine, Stanford, CA USA

**Keywords:** Genetic testing, Outcomes research, Genetic testing, Molecular medicine, Medical genomics

## Abstract

Whole-genome sequencing (WGS) is positioned to become one of the most robust strategies for achieving timely diagnosis of rare genomic diseases. Despite its favorable diagnostic performance compared to conventional testing strategies, routine use and reimbursement of WGS are hampered by inconsistencies in the definition and measurement of clinical utility. For example, what constitutes clinical utility for WGS varies by stakeholder’s perspective (physicians, patients, families, insurance companies, health-care organizations, and society), clinical context (prenatal, pediatric, critical care, adult medicine), and test purpose (diagnosis, screening, treatment selection). A rapidly evolving technology landscape and challenges associated with robust comparative study design in the context of rare disease further impede progress in this area of empiric research. To address this challenge, an expert working group of the Medical Genome Initiative was formed. Following a consensus-based process, we align with a broad definition of clinical utility and propose a conceptually-grounded and empirically-guided measurement toolkit focused on four domains of utility: diagnostic thinking efficacy, therapeutic efficacy, patient outcome efficacy, and societal efficacy. For each domain of utility, we offer specific indicators and measurement strategies. While we focus on diagnostic applications of WGS for rare germline diseases, this toolkit offers a flexible framework for best practices around measuring clinical utility for a range of WGS applications. While we expect this toolkit to evolve over time, it provides a resource for laboratories, clinicians, and researchers looking to characterize the value of WGS beyond the laboratory.

## Introduction

Whole-genome sequencing (WGS) is poised to exert a profound influence on clinical care by ushering individualized genomic medicine into routine practice. While technical and interpretive complexities remain, WGS is emerging as one of the most robust strategies for achieving timely diagnoses in undiagnosed rare disease populations^1–5^. However, for a diagnostic test such as WGS to be accepted into practice, commissioned in a health system, or receive coverage and reimbursement through health insurance, evidence of clinical utility and cost-effectiveness is generally required^6–8^. Unlike prospective clinical research where the ‘effectiveness’ of an intervention can be easily tied to a predefined health outcome, the concept of clinical utility in genetic medicine is rarely uniformly defined nor necessarily directly tied to a specific health outcome. As such, generating and evaluating evidence of clinical utility is complex. The challenge in defining clinical utility today is compounded by the extraordinary heterogeneity of rare diseases, as well as the polygenic nature of more common conditions for which WGS is expected to be relevant. In this paper, we aim to extend earlier conceptualizations of clinical utility as applied to the diagnostic use of WGS and suggest that this framework not only be used as a tool for evidence review^9–11^, but as a tool for measurement best practices. Our recommendations are intended for investigators, policy advisory bodies, payors, and health-care systems committed to providing value-based care and improving health and non-health related outcomes through the use of WGS at scale.

Early conceptualizations of clinical utility related to genetic testing emerged from work at the Centers for Disease Control^12^. The “ACCE” framework described analytical validity, clinical validity, clinical utility, and ethical implications as core components to evaluate before recommending genetic testing. Clinical utility was defined as the effect of genetic testing on “the balance of benefits and harms associated with the use of the test in practice, including improvement in measurable clinical outcomes and the usefulness or added value in decision-making compared with not using the test.” In the ACCE framework, a series of questions relating to test characteristics, health impacts, economic impacts, education, and implementation considerations are used to guide literature assessment^13^.

In the years that followed the development of the ACCE framework, scholars, professional groups, and payors continued to refine the dimensions and definitions of clinical utility. The Evaluation of Genomic Applications in Practice and Prevention (EGAPP) Working Group (EWG), for example, adapted a model proposed by Tatsioni et al.^14^, which itself was adapted from Fryback and Thornbury’s hierarchical model of efficacy for diagnostic tests^15^. In this model, the outcomes of interest for a test were organized into four groups: diagnostic and prognostic thinking, therapeutic choice, patient impact, and familial and societal impact. To organize and score the evidence reviewed, the EWG applied ACCE-framework questions to the individual domains of this model. More recently, the Association for Molecular Pathology proposed an expanded version of the ACCE model that attends to patient-centered definitions of clinical utility and aspects of clinical utility that extend beyond drug selection and associated health outcomes^11^ and the American College of Medical Genetics and Genomics (ACMG) defined clinical utility as the effect of a genetic test on diagnostic and therapeutic management, prognosis, health and psychological impacts on patients and their families as well as economic impacts on health-care systems^16^. The definitions of clinical utility offered by these organizations are similarly broad and align with other diagnostics-oriented evaluative frameworks suggested by Bossuyt et al.^17^, Williams et al.^18,19^, and the ClinGen Consortium^20^. However, even when a broad definition of clinical utility is invoked and dimensions identified, it is challenging to define the specific cascade of decisions, interventions, health and non-health related outcomes that might result from the information provided by a genomic test. In addition to the challenges associated with demonstrating benefit in a range of clinical contexts (i.e., prenatal, pediatric, and adult onset), assessing clinical utility requires attending to potential risks that may accompany diagnostic testing (e.g., misdiagnoses due to the wide range of test types, interpretive errors). Further, unintended consequences of secondary findings or unanticipated results from elective genomic testing^21^ could include overdiagnosis and unnecessary follow-up testing, monitoring, or labeling^22–24^. Given these complexities, we leverage an established conceptualization of clinical utility to offer a practical and specific approach to evidence collection for clinical utility. While the conceptualization of clinical utility that we offer is not novel, our emphasis on strategies for data collection extends existing frameworks that have been advanced for the primary purpose of evidence review.

## Recommendation development process

To attend to the complexities of evidence collection for clinical utility, we propose a measurement toolkit, comprised of a detailed measurement framework, indicators of utility, and suggested measurement strategies (Table 1, Supplementary Note 1). To establish this toolkit, the Medical Genome Initiative, a consortium of North American institutions aiming to expand access to high-quality clinical WGS through the publication of best practices, convened an expert panel. Members of the panel were limited to individuals with expertise in clinical genetics, laboratory genetics, and outcomes research; representatives from patients and families, research funders, and policy communities were not included. Through 10, 1-hour teleconference-based discussions over a 12-month period, we established a working definition of clinical utility and identified and debated conceptual frameworks that aligned with and helped to operationalize the working definition. Using a consensus-based process, we identified key measurement constructs, indicators, and data collection strategies aligned with each of the domains of the selected conceptual framework. Aligned with the EWG, we emphasize four domains considered to be central to the working definition of clinical utility. Further, we identified specific indicators within each domain to operationalize the meaning of each domain. We then identified key examples of empirical research that represent each domain of utility. Highlighting measurement and data collection strategies within identified examples, we developed a recommendation for advancing WGS research within each domain of clinical utility (Box 1). While the suggested approach can be applied to a range of genomic technologies and indications for testing, we focused on its application to WGS as a diagnostic tool for rare germline diseases. Where relevant, we address the elements of the framework that apply to identifying secondary findings in the context of indication-based WGS or risk-related findings in the context of elective WGS (Table 1).

**Table 1:** Application of the Fryback and Thornbury model of efficacy to genomic sequencing.

Fryback & Thornbury domain & definition	Measurement construct	Indicator	Data source	Data collection strategy (and examples)	References
Level 1: Technical efficacy In the laboratory, does the test measure what it purports to measure?	Analytic validity	Sensitivity (recall), specificity, precision (technical positive predictive value) when “gold standard” reference available Positive percent agreement (PPA), negative percent agreement (NPA) when only standard technical results available	Laboratory Information System or Lab report		Marshall^27^ FDA^81^
Level 2: Diagnostic accuracy efficacy Does the test result distinguish patients with and without the target disorder?	Clinical validity	Variant classification accuracy Genotype–phenotype matching Test outcome (positive, negative, inconclusive)	Laboratory Information System or Lab report		Rehm^38^ Strande^82^ Richards^83^
Level 3: Diagnostic thinking efficacy Does the test help a clinician to come to a diagnosis? Does the test change a clinician’s pretest estimate of the probability of a specific disease?	Understanding and refining disease etiology, prognosis, and relevant family history	Diagnostic classification (i.e., diagnosis, partial diagnosis, possible diagnosis, dual diagnosis)	Lab report, clinical consult notes	Primary data collection using case report forms, clinician checklists (e.g., Tool #1, Tool #2, Tool #3)	Scocchia^32^ Kingsmore^35^ Hayeems^40^
		Prognostic clarity	Clinical consult notes		
		Family history revised or refined	Pedigree, clinical consult notes	Primary data collection using case report forms, clinician checklists	French^30^
	Diagnostic investigation intensity	Use of additional diagnostic tests (lab, imaging, physiological) or consultations, avoidance or reduction of additional diagnostic tests	Lab report, clinical consult notes	Primary data collection using case report forms, clinician checklists (e.g., Tool #1, Tool #2, Tool #3, Tool #4)	Scocchia^32^ Kingsmore^35^ Hayeems^40^ PhenoTips Care Pathway v1.2
	Timeliness of diagnosis	Time to diagnosis	Lab report, clinical consult notes	Primary data collection using case report forms	Oei^34^ Kingsmore^35^
Level 4: Therapeutic efficacy Does the test aid in planning treatment? Does the test change or cancel planned treatment?	Medical management change^a^	Initiation, alteration, cessation of referral (e.g., sub-specialist or genetic counseling consultation) or intervention (e.g., medication, surgical procedure, surveillance; supportive care, cascade testing, educational service, rehabilitation resource, clinical trial, support group) for the index case	Medical records, clinical consult notes	Primary data collection using case report forms, clinician checklists (e.g., Tool #1, Tool #2, Tool #3, Tool #4, Tool #5)	Scocchia^32^ Kingsmore^35^ Hayeems^40^ PhenoTips Care Pathway v1.2 Niguidula^84^
Level 5: Patient outcome efficacy Do patients who undergo the test fare better than similar patients who do not?	Health-related outcomes^a^	Morbidity (burden of disease)	Medical records, Patient/Provider Administrative records	Primary data collection using case report forms or questionnaires Secondary analysis of data from administrative sources	Farnaes^4^ Schofield^85^
		Mortality (mortality rate, survival rate)	Medical records, Administrative records	Primary data collection using case report forms Secondary analysis of data from administrative sources	
		Quality of life^2^, functional health and well-being, phenotype-specific clinical outcomes	Patient or caregiver	Primary data collection using patient-reported outcome measures [e.g., Health Utility Index (HUI), PedsQL, SF-12 Health Survey, EQ5D, WHO QOL, WHO WOL-BREF, Bayley Scale of Infant and Toddler Development, Autism Diagnostic Observation Scale, Vineland Adaptive Behavior Scale]	HUI^86^ PedsQL^87^ EQ5D^88^ Bayley^44^ ADOS^45^ VABS^46^
		Health service utilization (e.g., number of outpatient visits, number of admissions, number of emergency visits, length of stay, rehospitalization rate, number of major medical procedures)	Medical records Administrative records	Primary data collection using case report forms Secondary analysis of data from administrative sources	Mitchell^69^
	Non-health-related outcomes^a^	Knowledge and understanding	Patient or caregiver	Primary data collection using patient-reported outcome measures (e.g., Genome Sequencing Knowledge Scale)	Kaphingst^54^
		Perceived personal utility		Primary data collection using patient-reported outcome measures (e.g., Perceived Personal Utility)	Lupo^55^ Kohler^52^
		Psychosocial well-being (e.g., empowerment, control, distress, uncertainty)		Primary data collection using patient-reported outcome measures (e.g., Empowerment: Genetic Counseling Outcome Scale (GCOS); Perceived Control: Perceived Personal Control Scale (PPC); Distress: Hospital Anxiety and Depression Scale (HADS), Impact of Events Scale (IES); Uncertainty: Tolerance for Ambiguity Scale (TFA), Perceived Uncertainty of Genomic Sequencing (PUGS))	GCOS^56^ PPC^57^ HADS^58^ IES^59^ TFA^60^ PUGS^61^
Level 6: Societal efficacy Is the test acceptable to society? Do cost benefit or cost-effectiveness analyses indicate that the test has efficacy at the health system or societal level?	Family, community, society impacts^a^	Family member risk identification, cascade testing, management changes (as per level 4) Patient/public values and preferences	Family members, members of the public, administrative records	Primary data collection using questionnaires, discrete choice experiments, patient or public engagement, qualitative methods Secondary analysis of data from administrative sources	Stark^89^ Marshall^90^ Kulchak Rahm^68^ Mitchell^69^ Lewis^71^ Chassagne^70^
	Value for money^a^	Direct costs: laboratory, clinician consultation interventions, health-care utilization	Payor fee schedules, laboratory price lists, administrative records	Primary data collection of health-care utilization using case report forms Secondary use of existing cost or utilization data (from medical records or administrative data)	Dragojlovic^91^ Tan^92^ Stark^95^ Schofield^85^ Tsiplova^93^ Yuen^94^ Schwarze^76^
		Indirect costs: productivity losses for index case and family members	Patient or caregiver or medical records	Patient or caregiver questionnaires	

^a^Applicable to measuring the clinical utility of secondary variants.

As articulated by Tatsioni et al.^14^ and the EWG^9^, Fryback and Thornbury’s model of efficacy presents a practical structure within which to operationalize the concept of clinical utility. The Fryback and Thornbury model, proposed in 1991 as a conceptual model for assessing the efficacy of diagnostic imaging, provides a hierarchical structure to assess medical tests at different levels of efficacy^15^. In this model, efficacy is defined as, “the probability of benefit to individuals in a defined population from a medical technology applied for a given medical problem under ideal conditions of use”^25^. Despite its reference to ideal conditions, Fryback and Thornbury concede overlap in meaning among the terms efficacy, effectiveness, and usefulness^15^, the latter of which apply to ordinary real-world settings that are germane to the question at hand. While other test evaluation frameworks have been well developed for genetic testing^26^, we were drawn to Fryback and Thornbury’s inclusion of the concept of diagnostic thinking as a core dimension of value, its comprehensiveness and consideration of varied perspectives from which value is considered (i.e., laboratory, diagnostician, clinical consultant, patient, society), its clear and simple language, and its application to any type of diagnostic technology.

**Fig. 1:**
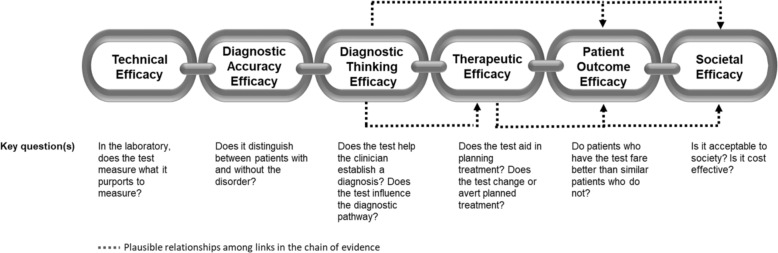
Clinical utility chain of evidence. Example outcomes assigned to clinical utility-related efficacy domains to demonstrate how these domains can be operationalized and measured. While the model is hierarchical, dotted lines between domains represent the notion that achieving a given level of efficacy is not always contingent upon the demonstration of efficacy at the preceding level.

The application of this model to WGS includes six levels of efficacy: technical efficacy, diagnostic accuracy efficacy, diagnostic thinking efficacy, therapeutic efficacy, patient outcome efficacy, and societal efficacy (Table 1, Fig. 1). The model is hierarchical; achieving a given level of efficacy is often but not always contingent upon a demonstration of efficacy at the preceding level. As described in Fig. 1, levels 1–3 are necessarily contingent but beyond level 3, a genetic test can achieve therapeutic, patient outcome, and/or societal impact in ways that are contingent upon one another or independent of one another. We retain the levels of technical and diagnostic accuracy efficacy (i.e., levels 1 and 2) as essential starting points in our guiding framework as they are fundamental precursors to achieving clinical utility. However, since these laboratory-based components of efficacy are well-debated and described in the WGS literature and in recent guidelines published by members of our group^27^, we focus here on four levels of the efficacy model (i.e., levels 3–6) that align most directly with a broad definition of clinical utility and extend beyond laboratory-based components of efficacy. In emphasizing these four levels of efficacy as components of clinical utility, our intent is to encourage the use of a broad set of health and non-health-related indicators of value to bolster the state of evidence in this area, rather than to convey that all aspects of clinical utility need to be achieved for WGS adoption and reimbursement.

Box 1 Recommendations for evaluating clinical utility of genomic testing

1. We recommend that assessment of clinical utility recognize four important dimensions: effects on diagnostic thinking, therapeutic management, patient health and non-health outcomes, and societal impacts.
2. We recommend that evaluation of clinical utility include assessment of impact on health care provider diagnostic thinking and decision-making. This assessment can include actively tracking changes in the differential diagnosis, influencing the diagnostic journey including decisions to use multiple genetic tests and various other diagnostic modalities, changes in prognostic certainty, and timeliness of the diagnostic information.
3. We recommend assessment of therapeutic efficacy include all medical recommendations and interventions that follow from a WGS result. While diagnostic results are more likely to lead to tailored recommendations/interventions, the absence of a pathogenic variant can enhance patient management decisions and should also be captured. Interventions can include therapies targeted to underlying disease mechanisms, supportive therapies, disease-specific monitoring plans, sub-specialist referrals, and any other resulting changes in management.
4. We recommend that assessment of clinical utility include evaluation of patient outcomes that may be directly and indirectly affected by the test. Patient outcomes can include health outcomes (e.g., hospitalization time, hospitalization events, morbidity and survival, disease-specific outcomes) and non-health outcomes (e.g., knowledge, psychosocial response, personal utility, decision quality).
5. We recommend that assessment of clinical utility include evaluation of societal effects that relate to family impacts, societal acceptability, and value for money. Benefits of information generated by WGS must be balanced against individual, community, and societal level costs and consequences.

## The Fryback and Thornbury model applied to evidence collection for WGS

### Diagnostic thinking efficacy (level 3)

Diagnostic thinking refers to the ways in which genomic testing may impact a clinician’s thinking and decision-making about the differential diagnosis they hold for a patient. Although the term ‘diagnosis’ is used very broadly to describe a wide range of laboratory, functional, physiological, and morphological abnormalities, we orient to the term insofar as it relates to identifying an underlying causal relationship between a genotype and an observed phenotype. Importantly, we operationalize diagnostic thinking efficacy as a construct that manifests at the level of the clinician, rather than at the level of the laboratory. An effect on diagnostic thinking, for example, could indicate that a test result strengthens or weakens an existing hypothesis about molecular etiology, or reassures the clinician by confirming a suspected diagnosis. We also include diagnostic investigation intensity and timeliness of diagnosis in this category. Checklists can be used to capture the way in which a test result alters a clinician’s diagnostic thinking, decision-making, and understanding of prognosis, while time to diagnosis and utilization or avoidance of additional diagnostic investigations can be captured by tracking dates of consultation, testing, and result reporting (Table 1). Physician report can be used as a core source for the former, while the latter can be accessed from electronic health records (Supplementary Note 1). Tracking the impact of WGS on diagnostic thinking and decision-making can occur when WGS is conducted at the beginning of a diagnostic journey as well as at subsequent points of re-analysis^28^. Here, we provide examples of how this aspect of utility is operationalized in the literature.

### Understanding disease etiology and prognosis

While meeting laboratory criteria for pathogenicity is necessary to establish a genetic diagnosis, the clinician’s interpretation of a variant in the context of an individual case is essential. For example, in a study that recruited 103 patients from pediatric sub-specialty clinics, variants that met established laboratory reporting criteria were reported in in 41% of patients^3^. In addition to meeting laboratory criteria, all candidate variants were discussed with the referring clinician and designated as diagnostic by laboratory and clinical consensus. Leveraging the clinicians’ deep knowledge of the patient’s phenotype, the study team was able to confirm that one patient had variants in two different genes contributing to her phenotype, two patients each had one variant that explained only a single aspect of a multisystem phenotype, and two patients were identified to each have a strong candidate variant that warranted further functional studies. Classified as dual, partial, and possible diagnoses, these findings highlight manifestations of diagnostic decision-making. Moreover, variants detected through WGS can prompt a deeper or more complex understanding of a patient phenotype. In a retrospective analysis^29^, 101 patients of 2076 (4.9%) who received a molecular diagnosis via exome sequencing, received a dual diagnosis. Through careful consideration of phenotypic and genotypic findings, some patients with dual molecular diagnoses were determined to have distinct phenotypes and some had overlapping phenotypes, wherein features could be attributable to either of the molecular diagnoses. Tracking diagnostic classifications such as these highlights how diagnostic thinking and decision-making relate to the nosology of disease, or the interplay between molecular diagnoses and the evolving spectrum of associated phenotypes. For future studies, incorporating clinician input into variant interpretation and explicitly tracking the nuanced clinical interpretation and classification of genomic variants would be examples of assessing this level of efficacy.

In the same way that variants detected by WGS can define and refine diagnostic thinking, WGS can define and refine prognostic thinking, or provide diagnostic clarity. In a study that performed trio WGS on a prospective cohort of families recruited in neonatal and pediatric intensive care units (NICU, PICU)^30^, a total of 195 families had WGS and 21% received a molecular diagnosis for the underlying genetic condition. Through medical record review, the authors highlighted the way in which the resolution of diagnostic and prognostic uncertainty informed four families’ decisions to proceed to supportive/palliative care.

Finally, while difficult to demonstrate in the empiric WGS literature to date, variants detected by WGS can bring new or existing phenotypic features into focus in an individual or family member that might not have been identified prior to testing. When this occurs in the context of either diagnostic or pre-dispositional testing, revisiting family histories for now relevant phenotypic features may reveal additional at-risk individuals or altered thinking about an inheritance pattern. For example, revised and enriched family histories were documented after disclosure of secondary WGS findings generated by the Clinical Sequencing Exploratory Research (CSER) Consortium^31^.

### Diagnostic investigation intensity

WGS results, particularly when negative or of uncertain clinical significance, may prompt the use of additional diagnostic tests with the aim of ruling out, or providing additional information to support a diagnosis in question. Suspicious findings may also prompt specialty consultations for deeper phenotyping. When WGS results reveal a diagnostic variant, other genetic or non-genetic tests, or more invasive diagnostic investigations (e.g., muscle biopsy), may be averted. For example, one study describes the use of WGS as a first-tier test in a dysmorphology clinic^32^, and reports that WGS identified molecular findings in 41 of 60 patients that were congruent with the reported phenotype. The authors tracked the ways in which the WGS results for some cases prompted additional diagnostic activities. These included gathering further phenotypic details from the family and initiating additional clinical work-up to further investigate the patient’s phenotype. In a cohort of 201 preschoolers with inherited eye disorders, medical records were reviewed to identify that unnecessary diagnostic tests were avoided in 21% of patients, as a result of genetic or genomic testing^33^.

### Timeliness of diagnosis

Recent work has demonstrated that WGS can achieve more timely diagnoses than conventional testing strategies. These studies demonstrate the capacity for WGS to end or avert diagnostic odysseys and to achieve a rapid turn-around time in urgent care settings. One study, for example, described the proportion of children enrolled in a complex care program with suspected genetic conditions and measured the testing period, types and costs of genetic tests pursued^34^. In a random sample of a retrospective cohort of 420 children, those with no genetic diagnosis underwent significantly more genetic tests than those with a confirmed genetic diagnosis [median interquartile range (IQR): six tests (4–9) vs. three tests (2–4), *p* = 0.002], more sequence-level tests and a longer, more expensive testing period than those with a genetic diagnosis [median (IQR): length of testing period: 4.12 years (1.73–8.42) vs. 0.35 years (0.12–3.04), *p* < 0.001; genetic testing costs C$8496 ($4399–$12,480) vs. C$2614 ($1605–$4080), *p* < 0.001]. Medical record reviews for data elements of this sort can describe the time (and resource) investment made in the diagnostic work-up of cases where WGS may be appropriate.

Approaching timeliness of diagnosis in a different way, a randomized controlled trial of the effectiveness of rapid WGS (rWGS) vs rapid exome sequencing (rES) was conducted with seriously ill infants with diseases of unknown etiology^35^. In addition to determining diagnostic performance of each test, the authors ascertained time to result by tracking key dates in the testing workflow (e.g., dates test ordered, sample accessioned, result reported). Time to result for rWGS and rES were similar (median 11.0 versus 11.2 days, respectively). However, time to result for ultra-rapid WGS was less (median 4.6 days, *p* < 0.0001).

### Therapeutic efficacy (level 4)

Therapeutic efficacy refers to the way(s) in which a genomic test result impacts a patient’s clinical trajectory of care beyond its impact on diagnosis. When a diagnostic variant has been identified with confidence, care plans may be tailored. For example, sub-specialist referrals, imaging or surveillance plans, targeted therapies or diet implications, surgical procedures or other types of acute medical management, supportive care, family member testing, or reproductive counseling may be initiated. While the outcome of the care plan decision is fundamental (as discussed in level 5), therapeutic efficacy focuses on the type and volume of care plan decisions that are directly attributable to the WGS result. When no variant or a variant of uncertain significance has been identified, care plans may be tailored towards more extensive diagnostic investigations (e.g., muscle biopsies, additional genetic analyses, family member testing), akin to that initiated in “influencing diagnostic pathway” above (Table 1). Checklists and case report forms can be used to extract these data from electronic medical records (i.e., clinician consult notes) or clinician surveys can be developed to capture this content from clinicians directly (Supplementary Note 1).

While not extensive, the literature that reflects on the therapeutic efficacy of WGS includes neonatal and pediatric intensive care, general pediatric, and adult medicine contexts. Most studies report prospective or retrospective case series or small cohorts. For example, WGS identified pathogenic variants in 48% of 130 children seen in a tertiary care, clinical genetics setting in China and collected data on therapeutic efficacy immediately post disclosure. Of the 62 diagnosed cases, active medical treatments were carried out in 30: 13 received transplantation (i.e., 2 liver transplants and 11 hematopoietic stem cell transplant), 17 received dietary or medicinal treatments, 20 received symptom treatment and referrals to rehabilitation, four received palliative care, and eight withdrew medical support^36^. Another prospective cohort study recruited 80 children with multiple congenital abnormalities and dysmorphic features and performed singleton ES^37^. Reflecting on therapeutic efficacy over a 12-month period, a clinical geneticist extracted information on changes in management, diagnostic investigations, tertiary pediatric hospital use, cascade testing in family members, and reproductive outcomes from medical records and from referring clinicians. While less common in the literature to date, some therapeutic efficacy studies use comparative or randomized study designs. For example, one pragmatic randomized controlled trial tested the hypothesis that rWGS increased the proportion of NICU and PICU infants receiving a genetic diagnosis within 28 days^2^. Short term clinical utility was assessed for those who received a molecular diagnosis by chart reviews and surveys with referring physicians; data collected included recommended instances of reproductive counseling, sub-specialty consults, medication alterations, procedures, and imaging. Furthermore, a recent meta-analysis defined clinical utility as the proportion of cases for whom there was a change in clinical management, excluding genetic counseling or reproductive planning, following WGS, ES, and microarray^1^. The clinical utility of WGS and ES was significantly higher than microarray (*p* < 0.0001).

Where feasible, to support robust data collection for therapeutic efficacy indicators, we recommend detailed collection of medical management recommendations attributable to all types of genetic test results (i.e., positive, negative, inconclusive^38^) in the immediate and longer term, and ascertainment of these data elements for the index patient and implicated family members. While data collection in this regard can prove challenging^39^, forms tailored to specific clinical contexts and index cases/family members as well as efforts to harmonize components of existing data collection tools that apply to most WGS settings are warranted. Examples of such tools are provided in Supplementary Note 1. As a complement or alternative to a harmonized case report form, an index that captures key aspects of diagnostic thinking and therapeutic efficacy from the clinician’s perspective has been developed^40^. Once validated, the Clinician-reported Genetic testing Utility InDEx (C-GUIDE), will quantify a test’s utility with respect to diagnostic thinking and therapeutic efficacy and will be usable across a range of clinical genetics settings (Supplementary Note 1).

### Patient outcome efficacy (level 5)

Improved health can be defined and measured in many different ways. Patient outcomes research enables a determination of the ways in which patients who receive a particular intervention (i.e., genomic test) fare in comparison to those who do not. While patient outcome-oriented research has been central to the practice of evidence-based medicine for years, its history in genomic medicine, with fairly recent developments in genotype-driven therapies, is not as well established. Where therapeutic impacts can be defined, measured, and attributed to genetic testing, traditional clinical effectiveness research in the context of rare disease should indeed proceed. In the absence of direct links between genetic test results and traditional health-related outcomes, however, we align our clinical utility toolkit to current thinking about health technology assessment in genomic medicine. Precisely because genome diagnostics are not always tied directly to health outcomes, non-health-related patient outcomes are gaining traction among health economists and decision-makers^41,42^. As such, we have divided the wide range of plausible patient outcomes into two core categories: (i) health-related and (ii) non-health-related (Table 1).

Health-related outcomes can be characterized by a wide range of indicators related to morbidity, mortality, quality of life (i.e., functional health and well-being), intensity of symptoms, and intensity of health service utilization. Broad measures of this sort can be applied in the aggregate to a group of rare conditions or to any specific disease context, regardless of rarity, and are often the most feasible starting point for evaluating WGS in cohorts for which diagnoses are unknown and specific medical outcomes cannot be anticipated a priori. The World Health Organization has provided guidance on the assessment of health and disability in children and youth through its development of the International Classification of Functioning, Disability and Health (ICF Checklist; https://www.who.int/classifications/icf/icfchecklist.pdf?ua=1)^43^. While we recommend that such measures be incorporated into outcomes-oriented studies in genomics when possible to define, we note that many features of rare diseases are not well captured by these measurement instruments and that additional measures tailored to specific phenotypes, phenotype categories, or clinical settings warrant consideration (e.g., Bayley Scale of Infant and Toddler Development^44^, the Autism Diagnostic Observation Scale^45^, the Vineland Adaptive Behavior Scale^46^). While case reports and a small number of controlled comparative studies present clinical outcomes associated with WGS and/or genotype-directed therapies^47–49^ and more robust observational studies are underway^50^, measurement strategies that enable researchers to attribute health-related outcomes to WGS remain under-developed.

To mitigate this measurement gap, some WGS study teams have opted to assess medical benefit according to expert opinion. For example, one study assessed patient outcome efficacy in a retrospective cohort study of acutely ill infant inpatients^4^. When a diagnosis was identified, the authors explicitly considered what might have happened if WGS had not been performed. They presented alternate care maps to an expert Delphi panel for review. They then described specific changes in medical or surgical treatment that occurred as a result of molecular diagnoses in 13 (31%) of 42 infants receiving rWGS. These included initiation of a medication in a child with megacystis-microcolon-intestinal hypoperistalsis syndrome which improved gut motility, avoidance of a liver transplantation in child with a *JAG1* deletion, avoidance of severe intellectual disability, and the avoidance of risks of death waiting for a transplant or pancreatic surgery in other patients. In addition, many invasive procedures were avoided. rWGS was judged to have prevented morbidity in 11 (61%) of 18 diagnosed infants, compared with none by standard of care. While this approach allowed a specific determination of avoided morbidity compared with reference cases known to have the same diagnosis, it required intensive review of medical records by experts, the formation of a Delphi panel, and hypothetical judgments. That said, this approach has been replicated in other clinical presentations^51^ and appears to be feasible for small, heterogeneous, rare disease cohorts.

Attending to non-health related outcomes presents another strategy for mitigating the limitations of traditional health outcomes in the context of genomics. Standard quality of life measures (e.g., the EuroQol five-dimensional [EQ5D] questionnaire https://euroqol.org/), used to generate quality-adjusted life years (QALYs), for example, do not consider the non-health impacts of genome diagnostics^41^. While empiric examples are emerging in the WGS literature that invoke traditional measures of QALYs^37^, health economists have questioned whether the QALY—a health outcome focused metric—can properly quantify the outcomes that are important to patients undergoing genetic testing. While some WGS applications may improve health outcomes, most currently generate non-health outcomes. As such, we recommend that assessments of patient outcome efficacy include non-health-related outcomes regarding patient knowledge, personal utility, and psychosocial well-being (Table 1^52–59^). With respect to psychological response to WGS, a meta-analysis of data collected from multiple sites provides a helpful example^60^. Specifically, the study team assessed state anxiety, depressive symptoms, and multidimensional test-related outcomes following the return of WGS results in a range of clinical cohorts. State anxiety and depressive symptoms were measured pre- and post-WGS results disclosure using the Hospital Anxiety and Depression Scale (HADS)^61^, the Personal Health Questionnaire 9-item (PHQ-9)^62^, and the Generalized Anxiety Disorder 7-item (GAD-7)^63^. The multidimensional impact of receiving WGS results was measured following results disclosure using a modified version of the Multidimensional Impact of Cancer Risk Assessment (MICRA)^64^ or the Feelings About genomiC Testing Results (FACToR)^65^. To ascertain psychosocial response over time, post-disclosure surveys were administered at 6 weeks and 6 months following WGS disclosure. Aligned with this example, we recommend ascertaining non-health outcomes with validated tools where possible, defining by whom (e.g., patients, providers, caregivers), how (self-administered vs research team administered), when (i.e., baseline and follow-up) survey instruments are administered, and to what result disclosure process participants are responding (i.e., in-person vs. telephone-based consultation, genetics professional-led vs. non-genetics professional-led consultation). When identifying non-heath outcome measures, attending to literacy level and languages in which an outcome measure is available warrant consideration.

### Societal efficacy (level 6)

Finally, societal efficacy refers to the societal acceptability of WGS, broadly speaking. It poses questions about whether the cost of a genetic test in a particular clinical context is acceptable—to society as a whole—even though individuals (rather than whole populations) may benefit. It asks about the limits and contexts of appropriate use of WGS and the financial and ethical trade-offs required. For the purpose of our proposed toolkit, we orient to societal efficacy in two primary ways: (i) the impact of WGS on individuals or groups of individuals that extend beyond the index case and (ii) the value of WGS relative to its cost.

First, individuals that extend beyond the index case for whom WGS may have impacts include family members, defined communities or target populations, and society as a whole. A wide range of strategies can be used to ascertain data on impacts of this kind. For research questions operating at the level of the family unit, indicators of utility may include whether or not family members have been identified to be at risk for a heritable condition, whether they have pursued genetic counseling, testing, and/or surveillance and whether reproductive decision-making has been influenced by family-based WGS. Both short and long-term health and non-health outcomes associated with cascade family testing warrant consideration. Data collection strategies may include medical record review or survey administration. Patient- and family-level data can be compared to clinical practice guidelines as a strategy for gauging alignment with clinical standards^66^. One study, for example, used a combination of medical record review and parental surveys to collect cascade testing and parental reproductive outcomes triggered by exome sequencing of 80 infants^37^. Of 88 eligible first-degree relatives, 79 were tested for 52 variants. Of these, 12 relatives received a molecular diagnosis and two received a change in medical management. In addition, 16 couples sought advice from a pre-implantation or prenatal genetics service. Indeed, this conceptualization of societal efficacy overlaps with our conceptualization of therapeutic efficacy. In this domain, familial implications are deliberately in focus whereas in level 4, familial implications may or may not be considered to be in scope.

For research questions operating at the community or population level, societal efficacy can be informed by a range of quantitative and qualitative patient and public engagement strategies. Specific examples of these include discrete choice experiments (DCE), deliberative dialog, and empirical ethics (Table 1). In a DCE, preferences for attributes of a technology are enumerated by analyzing participants’ responses to a series of choice tasks. In a study that examined how parents and families value exome sequencing, a DCE was constructed to include 14 choice tasks with six attributes, each of which was characterized by three levels. A statistical model was constructed to estimate participants’ willingness to pay, willingness to wait for test results, and minimum acceptable chance of a diagnosis for changes in each attribute. DCE modeling is a powerful approach for quantifying how characteristics of a technology are differentially valued by members of society.

Public deliberation presents another strategy for ascertaining societal impacts. It is based on the premise that many of the important decisions faced by a society—particularly those that involve competing values and complex trade-offs in health care—are best made by decision-makers in partnership with a public that has had the opportunity to be educated about and deliberate an issue^63,65,67,68^. For example, one study used a deliberative approach to ascertain parental attitudes related to returning medically actionable variants in healthy children who were sequenced as part of a population biobank. Following an educational session that presented key ethical tensions and related professional guidelines, focus group participants were asked to respond to ‘real life’ media stories that portrayed the issues as a way of eliciting values and preferences. Qualitative thematic analysis identified participant-derived educational and process strategies for biobanks tasked with navigating the ethics of identifying a range of WGS result types. Similarly, when parents of children in a research biobank were offered choices about return of various categories of WGS results, then provided with lists of what they would be missing through those choices, they altered their perspective about the value of such information, opting for more information rather than less^69^. Rich insights and lived experiences related to the acceptability of WGS can also be elicited from non-deliberative qualitative data collection strategies (Table 1)^70,71^. Like quantitative methods, specific skillsets and strategies for optimizing study design and rigor are required.

Finally, societal efficacy refers to the value of WGS relative to its cost. While it is beyond the scope of our expertise to recommend specific approaches to economic evaluation, we point the reader to a growing literature that reflects on the challenges of traditional (QALY-based) approaches to economic evaluation (e.g., cost-effectiveness analysis, cost-utility analysis, cost–benefit analysis) in the context of genomic medicine^41,72,73^. While non-health related outcomes for genomic medicine (as discussed in level 5) require further development and validation, their integration into economic evaluations and health technology assessment is gaining traction^74^. For example, the Second Panel on Cost Effectiveness in Health and Medicine (an update on the Panel on Cost-Effectiveness in Health and Medicine convened by the US Public Health Service in 1993)^75^ noted that decision-makers need a “quantification and valuation of all health and non-health effects of interventions”^74^. While efforts to define and measure the health effects of WGS remain essential, defining and measuring the non-health effects, as articulated in toolkit levels 3–5, align with current thinking and emerging practice of health technology assessment and decision-making entities deliberating the value of genomic medicine internationally^41,72,73,76–78^.

## Summary and future directions

Despite the demonstrated technical superiority of WGS as a diagnostic tool for rare disease compared to conventional genetic testing^1–5^, characterizing the full value of this technology—in ways that are accessible to health system decision-makers—poses conceptual and operational challenges. Drawing upon the Fryback and Thornbury hierarchical model of efficacy and expert opinion, we offer a refined conceptual framework that attends to the dimensions of clinical utility for genomic medicine. Operationalizing each dimension of utility to include specific indicators, examples, and measurement strategies, we provide a resource to the genomics research community invested in generating evidence that will guide efforts to optimize the patient, provider, and system-level value of WGS. In our view, the tools developed by Socchia et al.^32^, Kingsmore et al.^35^, and Hayeems et al. (C-GUIDE)^40^ represent comprehensive strategies for attending to diagnostic thinking efficacy and therapeutic efficacy. The intent of the C-GUIDE was precisely to establish a standardized and validated approach to collecting data on these aspects of clinical utility. Once validated, we encourage broad use of this tool. Since patient outcome efficacy and societal efficacy can be addressed in multiple ways, we encourage careful selection of study design, data sources, and existing validated measures (Table 1) to address these dimensions of clinical utility. In providing this resource, our intent is not to impose a threshold for what constitutes sufficient clinical utility, as this depends on which stakeholder is seeking such evidence. Clinicians, patients, and payers may define, weigh, and balance this type of evidence differently. Rather, our intent is to equip our colleagues with an organized way of thinking about generating evidence of this kind and a starting point for a set of strategies for doing so. While we have not focused on the relative strengths and limitations of various study designs or data sources, we encourage our colleagues to consider traditional hierarchies of evidence^79^ and to embed the proposed data collection concepts and strategies into study designs that are optimized for the research question posed. Where possible, we encourage the use of prospective, comparative approaches. We also encourage integrating studies focused on clinical utility concurrent with early translation of WGS in clinical care by inviting patients/families for whom WGS is indicated to participate in such studies at the time they are offered testing. This will facilitate the ascertainment of short- and long-term outcomes related to clinical utility and earlier knowledge translation to decision maker partners. Implementation-effectiveness hybrid designs are particularly well suited to this context^80^.

Finally, we offer the diagnostic application of WGS for rare germline disease across pediatric and adult medicine settings as a starting point, however, we anticipate and encourage modifications to this framework as further applications of WGS and other -omic technologies evolve. Moreover, since the development of this approach was informed by clinical and laboratory genetics expertise, we welcome input from members of the patient, policy, and research funding communities to guide our thinking on an updated version. While study contexts will invariably differ and require tailored designs and measures, we encourage where feasible, the use of common tools for measuring clinical utility. To our knowledge, uniform data collection tools of this kind are not currently in use; they warrant development, validation, and open sharing. Where harmonization and collaboration are possible, a more immediate and robust evidence base can be established to inform patient, clinician, policy, and payor decisions, in turn improving opportunities for equitable and sustainable access to high-quality WGS.

## Supplementary information

Supplementary Information

## Data Availability

All data generated or analyzed during this study are included in this published article (and its supplementary information files).
