# Supplementary material for: Clinical utility of genomic sequencing: a measurement toolkit
Source: NPJ Genom Med. 2020 Dec 15;5:56. doi: 10.1038/s41525-020-00164-7 (PMC7738524; doi:10.1038/s41525-020-00164-7)
Supplement: Supplementary file 1 — Supplementary Information [file 41525_2020_164_MOESM1_ESM.pdf]

SUPPLEMENTARY MATERIALS

Supplementary Note 1: Clinical Utility Measurement Tools (Examples from the literature)

Tool #1: Clinical Utility Form (Scocchia et al. 2019)

Tool #2: Clinician Assessment Form (Kingsmore et al. 2019)

Tool #3: C-Guide Version 1.1 (Hayeems et al. 2019)

Tool #4: PhenoTips Care Pathway Tool v1.2 (PhenoTips.com)

Tool #5: Clinician Checklist (Niguidula et al. 2018)

Tool #1: Clinical Utility Form (Scocchia et al. 2019[1])

iHope **Barcode Number:**

Clinical Utility Form

1. Diagnoses prior to submission of sample for whole genome sequencing (WGS):

2. Diagnoses after receipt of WGS

3. WGS (please select the best response):

☐ Confirmed the clinical diagnosis

☐ Produced a new diagnosis

☐ Gave a possible diagnosis

☐ Ruled out a suspected diagnosis

☐ Did not contribute to the diagnosis

☐ Other, please specify:

☐ NA

4. If WGS confirmed or produced a diagnosis, was confirmatory testing then performed?

☐ YES

☐ NO

☐ NA

5. Did WGS change the management of the patient?

☐ YES

☐ NO

☐ NA

If no, skip to question 6. If yes, mark all that apply:

☐ Condition specific management (specific therapy targeted to the

diagnosis) Specify therapy:
Specify therapy:
Specify therapy:

☐ Condition Specific Supportive Interventions (supportive care informed by a better understanding of the diagnosis)

Specify supportive care:
Specify supportive care:
Specify supportive care:

☐ Palliative or End of Life Care (palliative care informed by an improved understanding of the prognosis) Specify type of palliative care:
Specify type of palliative care:
Specify type of palliative care:

6. Did the WGS result change the post genetic consultation?

☐ YES If YES, describe how?

☐ NO

☐ NA

7. The results of the WGS led to additional clinical testing (check all that apply)

☐ Specialty consultation	Specify:
☐ Physiological testing	Specify:
☐ Imaging	Specify:
☐ Laboratory	Specify:
☐ None	
☐ NA	

8. Please provide any additional detail:

Tool #2: Clinician Assessment (72-hours) Form (Kingsmore et al 2019[2])

Clinician Assessment
(72-Hours)

Clinician Credentials (MD, NNP, PA, etc.): ______________________________ Years of Experience of Clinician: ______________________________________

Clinical Utility (select all that apply)

- ◻ None
- ◻ Neutral

Diagnostic Utility:

- ◻ Diagnosis avoided complications
- ◻ Targeted treatment improved long-term outcomes
- ◻ Having a name for the condition was helpful
- ◻ Better understanding of pathophysiology of disorder
- ◻ Improved communication of outcomes/expectations with families
- ◻ Diagnosis caused more stress and/or confusion with family
- ◻ Diagnosis caused more confusion among clinical staff
- ◻ Family planning counseling initiated
- ◻ Further testing not necessary, additional testing required
- ◻ Diagnosis not fully understood at this time
- ◻ Other: ____

Change in clinical management:

- ◻ Surgical intervention added
- ◻ Surgical intervention removed
- ◻ Medication added
- ◻ Medication removed
- ◻ Medication changed
- ◻ Diet changed
- ◻ New specialty service sought
- ◻ New specialty service no longer required
- ◻ New imaging sought
- ◻ Prior imaging cancelled
- ◻ New test ordered
- ◻ Prior testing cancelled
- ◻ Screening for additional comorbidities added
- ◻ Screening for additional comorbidities removed
- ◻ Palliative care initiated
- ◻ Palliative care withdrawn
- ◻ Other: ____

Other care changes:

- ◻ Clinical testing of family members recommended
- ◻ Enrollment of family members into research study
- ◻ Patient eligible for new research study
- ◻ Other: ____

CONFIDENTIAL

Tool #3: C-Guide (Hayeems et al. 2019[3])

Undergoing validation

Please contact the developer prior to use (robin.hayeems@sickkids.ca)

Copyright 2020, THE HOSPITAL FOR SICK CHILDREN

C-GUIDE Version 1.1

The Clinician-reported Genetic testing Utility InDEx (C-GUIDE)™ aims to capture the clinical utility of genetic testing once results are disclosed, from the perspective of the ordering clinician.

Thinking about the primary result you just disclosed, please complete the following:

N.B. If you disclosed multiple results, please complete the C-GUIDE once for each result disclosed. You will be prompted to do this after you complete C-GUIDE for the first result. If you disclosed secondary or pharmacogenomics results, you will be asked about those specific results later.

Item	Response Options
The genetic testing that my patient had…	
1. Provided a genetic explanation for my patient's health condition	☐ Provided a COMPLETE genetic explanation [2]<br>☐ Provided a PARTIAL genetic explanation [1]<br>☐ Provided a POSSIBLE genetic explanation [1]<br>☐ Provided NO genetic explanation [0]
2. Reduced the likelihood of other differential diagnoses	☐ COMPLETELY REDUCED the likelihood of other differential diagnoses [2]<br>☐ PARTIALLY REDUCED the likelihood of other differential diagnoses [1]<br>☐ DID NOT REDUCE the likelihood of other differential diagnoses [0]
3. Provided information about the natural history of or medical issues associated with my patient's condition	☐ Provided SIGNIFICANT information about the natural history of or medical issues associated with my patient's condition [2]<br>☐ Provided SOME information about the natural history of or medical issues associated with my patient's condition [1]<br>☐ Provided NO information about the natural history of or medical issues associated with my patient's condition [0]
4. Indicated that further testing to identify a genetic diagnosis can be avoided	☐ Indicated that further testing to identify a genetic diagnosis CAN BE AVOIDED [2]<br>☐ Indicated that further testing to identify a genetic diagnosis MAY STILL BE REQUIRED, now or in the future [0]
5. Indicated that previous surveillance or monitoring related to my patient's condition can be discontinued or avoided	☐ Indicated that previous surveillance/monitoring can be DISCONTINUED OR AVOIDED [2]<br>☐ Indicated that previous surveillance/monitoring is STILL REQUIRED [0]<br>☐ Previous surveillance/monitoring is NOT RELEVANT to this case [0]
6. Facilitated my patient's access to or continuation of a community or educational	☐ FACILITATED access to or continuation of a community or educational service [2]

service (e.g. learning, rehabilitation resources) that would not have been available without the testing	☐ DID NOT FACILITATE access to or continuation of a community or educational service [0]
7. Enabled me to identify and access a clinical trial that I wouldn't have been able to access without the testing	☐ ENABLED me to IDENTIFY and ACCESS a clinical trial [2]<br>☐ ENABLED me to IDENTIFY a clinical trial [1]<br>☐ DID NOT ENABLE me to identify or access a clinical trial [0]
8. Enabled me to identify a support group for my patient or his/her family that I wouldn't have considered without the testing	☐ ENABLED me to identify a support group [2]<br>☐ DID NOT ENABLE me to identify a support group [0]
9. Prompted a referral or investigation for the purpose of surveillance or monitoring that would not have been prompted on clinical grounds	☐ PROMPTED a referral or investigation for surveillance/monitoring [2]<br>☐ PROMPTED a referral or investigation for surveillance/monitoring that MAY NOT BE NECESSARY (e.g. variant of uncertain significance) [1]<br>☐ DID NOT PROMPT a referral/investigation for surveillance/monitoring [0]
10. Provided information to guide medication management	☐ GUIDED current medication management [2]<br>☐ MAY GUIDE medication management in the future [1]<br>☐ DID NOT PROVIDE information that would guide medication management, now or in the future [0]
11. Provided information about surgical management	☐ ENABLED a discussion or offer of a surgical option [2]<br>☐ AVOIDED a discussion or offer of a surgical option [1]<br>☐ A surgical option is NOT RELEVANT at this time or NOT RELATED to the genetic test results [0]
12. Provided information about a contraindicated behaviour (e.g. competitive sports)	☐ ENABLED me to provide information about a contraindicated behaviour [2]<br>☐ Information about a contraindicated behaviour is NOT RELEVANT at this time [0]
13. Provided recurrence risk information for my <u>patient</u>	☐ Provided recurrence risk information that is RELEVANT to my patient at this time [2]<br>☐ Provided recurrence risk information that MAY BE RELEVANT to my patient in the future [1]<br>☐ Cannot be determined (e.g. variant of uncertain significance, did not provide information) [0]
14. Provided recurrence risk information for my <u>patient's family</u>	☐ Provided recurrence risk information that is RELEVANT to my patient's family at this time [2]<br>☐ Provided recurrence risk information that MAY BE RELEVANT to my patient's family in the future [1]<br>☐ Cannot be determined (e.g. variant of uncertain significance, family member(s) did not receive testing or unknown if tested) [0]

15. Clarified potential health risks for my patient's family	☐ CLARIFIED potential health risks for my patient's family [2]<br>☐ DID NOT CLARIFY health risks for my patient's family [0]<br>☐ Cannot be determined (e.g. variant of uncertain significance, family member(s) did not receive testing or unknown if tested) [0]
16. Generated psychosocial benefit for my patient or his/her family	☐ SIGNIFICANT psychosocial benefit was experienced [2]<br>☐ MODERATE psychosocial benefit was experienced [1]<br>☐ NO psychosocial benefit was experienced [0]<br>☐ Cannot be determined [0]
17. Generated psychosocial concern for my patient or his/her family	☐ SIGNIFICANT psychosocial concern was experienced [-2]<br>☐ MODERATE psychosocial concern was experienced [-1]<br>☐ NO psychosocial concern was experienced [0]<br>☐ Cannot be determined [0]

C-GUIDE: Secondary Variants

Did you disclose SECONDARY variant results?

N.B. For the purpose of this index, secondary variants include medically actionable variants unrelated to the indication for testing.

- ☐ Yes
- ☐ No

If yes, please complete a C-GUIDE once for each secondary result disclosed.

**Item**	**Response options**
The genetic testing that my patient had…	
1. Prompted a referral or investigation for the purpose of surveillance or monitoring that would not have been prompted on clinical grounds	☐ PROMPTED a referral or investigation for surveillance/monitoring [2]<br>☐ PROMPTED a referral or investigation for surveillance/monitoring that MAY NOT BE NECESSARY (e.g. variant of uncertain significance) [1]<br>☐ DID NOT PROMPT a referral/investigation for surveillance/monitoring [0]
2. Provided information to guide medication management	☐ GUIDED current medication management [2]<br>☐ MAY GUIDE medication management in the future [1]<br>☐ DID NOT PROVIDE information that would guide medication management, now or in the future [0]
3. Provided information about surgical management	☐ ENABLED a discussion or offer of a surgical option [2]<br>☐ AVOIDED a discussion or offer of a surgical option [2]<br>☐ A surgical option is NOT RELEVANT at this time or NOT RELATED to the genetic test results [0]
4. Provided information about a contraindicated behaviour (e.g. competitive sports)	☐ ENABLED me to provide information about a contraindicated behaviour [2]<br>☐ Information about a contraindicated behaviour is NOT RELEVANT at this time [0]
5. Provided recurrence risk information for my patient	☐ Provided recurrence risk information that is RELEVANT to my patient at this time [2]

	☐ Provided recurrence risk information that MAY BE RELEVANT to my patient in the future [1]<br>☐ Cannot be determined (e.g. variant of uncertain significance, did not provide information) [0]
6. Provided recurrence risk information for my patient's family	☐ Provided recurrence risk information that is RELEVANT to my patient's family at this time [2]<br>☐ Provided recurrence risk information that MAY BE RELEVANT to my patient's family in the future [1]<br>☐ Cannot be determined (e.g. variant of uncertain significance, family member(s) did not receive testing or unknown if tested) [0]
7. Clarified potential health risks for my patient's family	☐ CLARIFIED potential health risks for my patient's family [2]<br>☐ DID NOT CLARIFY health risks for my patient's family [0]<br>☐ Cannot be determined (e.g. variant of uncertain significance, family member(s) did not receive testing or unknown if tested) [0]
8. Generated psychosocial benefit for my patient or his/her family	☐ SIGNIFICANT psychosocial benefit was experienced [2]<br>☐ MODERATE psychosocial benefit was experienced [1]<br>☐ NO psychosocial benefit was experienced [0]<br>☐ Cannot be determined [0]
9. Generated psychosocial concern for my patient or his/her family	☐ SIGNIFICANT psychosocial concern was experienced [-2]<br>☐ MODERATE psychosocial concern was experienced [-1]<br>☐ NO psychosocial concern was experienced [0]<br>☐ Cannot be determined [0]

C-GUIDE: Pharmacogenomic Results

Did you disclose PHARMACOGENOMIC results?

N.B. For the purpose of this index, pharmacogenomic results include those that are identified through a targeted pharmacogenomic analysis and could be relevant to medication management now or in the future.

- ☐ Yes
- ☐ No

If yes, please complete C-GUIDE once for the pharmacogenomic result(s) disclosed. For the purpose of this study, pharmacogenomic results are typically disclosed as a 'cluster' of variants.

**Item**	**Response options**
The genetic testing that my patient had…	
1. Provided information to guide medication management for my patient	☐ GUIDED current medication management [2]<br>☐ MAY GUIDE medication management in the future [1]<br>☐ DID NOT PROVIDE information that would guide medication management, now or in the future [0]
2. Provided information about medication management for my patient's family	☐ GUIDED current medication management for my patient's family [2]<br>☐ MAY GUIDE medication management for my patient's family in the future [1]<br>☐ DID NOT PROVIDE medication management information for my patient's family, now or in the future [0]<br>☐ Cannot be determined (e.g. variant of uncertain significance, family member(s) did not receive testing or unknown if tested) [0]
3. Generated psychosocial benefit for my patient or his/her family	☐ SIGNIFICANT psychosocial benefit was experienced [2]

	☐ MODERATE psychosocial benefit was experienced [1]<br>☐ NO psychosocial benefit was experienced [0]<br>☐ Cannot be determined [0]
4. Generated psychosocial concern for my patient <u>or</u> his/her family	☐ SIGNIFICANT psychosocial concern was experienced [-2]<br>☐ MODERATE psychosocial concern was experienced [-1]<br>☐ NO psychosocial concern was experienced [0]<br>☐ Cannot be determined [0]

Tool #4: PhenoTips Care Pathway *v1.2*[4] - Post Test Medical Management Impact
Developed by Hayeems et al and https:///phenotips.com

Sample Components

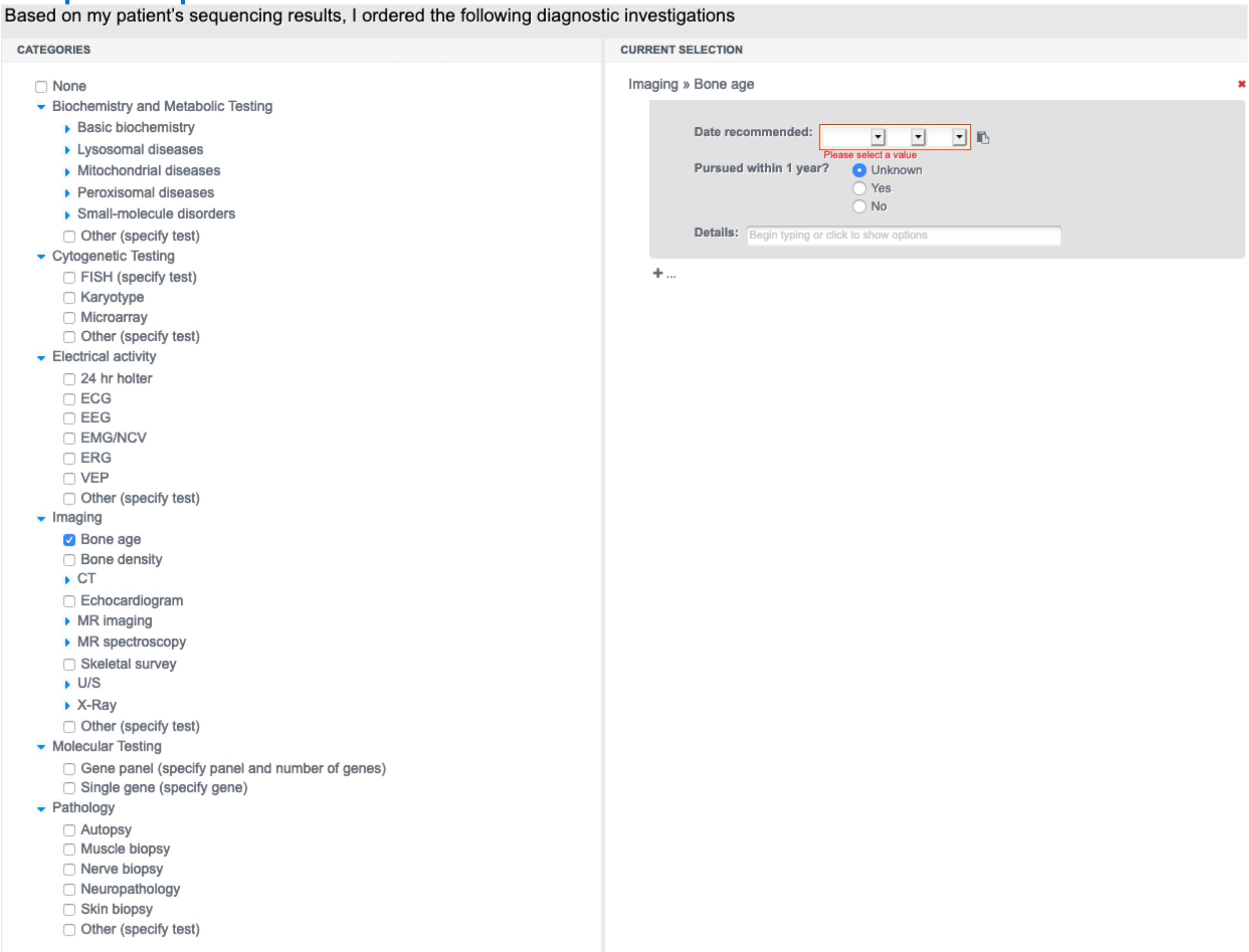

Based on my patient's sequencing results, I did not need to order the following diagnostic investigations

CATEGORIES

- [ ] No diagnostic investigations were avoided
- Biochemistry and Metabolic Testing
  - Basic biochemistry
  - Lysosomal diseases
  - Mitochondrial diseases
  - Peroxisomal diseases
  - Small-molecule disorders
  - [ ] Other (specify test)
- Cytogenetic Testing
  - [ ] FISH (specify test)
  - [ ] Karyotype
  - [ ] Microarray
  - [ ] Other (specify test)
- Electrical activity
  - [ ] 24 hr holter
  - [ ] ECG
  - [ ] EEG
  - [ ] EMG/NCV
  - [ ] ERG
  - [ ] VEP
  - [ ] Other (specify test)
- Imaging
  - [ ] Bone age
  - [ ] Bone density
  - CT
  - [ ] Echocardiogram
  - MR imaging
  - MR spectroscopy
  - [ ] Skeletal survey
  - U/S
  - X-Ray
  - [ ] Other (specify test)
- Molecular Testing
  - [ ] Gene panel (specify panel and number of genes)
  - [ ] Single gene (specify gene)
- Pathology
  - [ ] Autopsy
  - [x] Muscle biopsy
  - [ ] Nerve biopsy
  - [ ] Neuropathology
  - [ ] Skin biopsy
  - [ ] Other (specify test)

CURRENT SELECTION

Pathology » Muscle biopsy ✖

Details: Begin typing or click to show options ✖

+ ...

Based on my patient's sequencing result, I recommended the following care plan for my patient:

CATEGORIES	CURRENT SELECTION

MONITORING AND LONG TERM CLINICAL MANAGEMENT

- [ ] No monitoring or long term management recommended

Care Team

- Allied health care referral
- Subspecialist referral

Surveillance Management

- Biochemistry and Metabolic Testing
- Electrical activity
- Imaging

ACTIVE MANAGEMENT

- [ ] No active management recommended
- Changes to medication(s) treating symptoms
- Disease-specific therapy
  - [ ] Special diet
  - Targeted drugs for specific RD
    - [x] Enzyme replacement therapy
    - [ ] Small molecule therapy
    - [ ] Other
  - [ ] Vitamin or mineral supplementation
  - [ ] Other
- [ ] Invasive Procedure (e.g. defibrillator)

RESEARCH OPPORTUNITIES TRIGGERED BY THE RESULT

- [ ] No research activities triggered
- [ ] Clinical trial
- [ ] Disease mechanism study
- [ ] Disease registry
- [ ] Natural history study

CURRENT SELECTION

Disease-specific therapy » Targeted drugs for specific RD » Enzyme replacement therapy

Date recommended: Please select a value

Pursued within 1 year? (•) Unknown ( ) Yes ( ) No

Details: Begin typing or click to show options — Please enter a value

+ ...

Based on my patient's secondary sequencing result, I recommended the following care plan for my patient:

CATEGORIES	CURRENT SELECTION

MONITORING AND LONG TERM CLINICAL MANAGEMENT

- [ ] No monitoring or long term management recommended

Care Team

- Allied health care referral
- Subspecialist referral

Surveillance Management

- Biochemistry and Metabolic Testing
- Electrical activity
- Imaging
  - [ ] Bone age
  - [ ] Bone density
  - CT
  - [x] Echocardiogram
  - MR imaging
  - MR spectroscopy
  - [ ] Skeletal survey
  - U/S
  - X-Ray
  - [ ] Other (specify test)

CURRENT SELECTION

Imaging » Echocardiogram

Date recommended: Please select a value

Pursued within 1 year? (•) Unknown ( ) Yes ( ) No

Details: Begin typing or click to show options

+ ...

FAMILY IMPLICATIONS

Number of 1st degree relatives counselled:

Prenatal counselling: Unknown

Non-prenatal counselling: Unknown

Number of 2nd degree relatives counselled:

Prenatal counselling: Unknown

Non-prenatal counselling: Unknown

Number of 1st degree relatives tested:

Prenatal testing: Unknown

Non-prenatal testing: Unknown

Number of 2nd degree relatives tested:

Prenatal testing: Unknown

Non-prenatal testing: Unknown

Tool #5: Clinician Checklist (Niguidula et al. 2018[5])

Each person who completes the survey will be entered into a drawing to win a $100 gift card to Amazon.com. Thank you in advance for your participation! Please take a few moments to provide us with some brief information

Specifically in reference to this patient's case. The goal of this research study is to determine how, or if, the results of exome sequencing affect patients' clinical management. The responses from this survey will be linked to your patient's information so that we can account for the many variables between different patients' indications, clinical features, and results. No identifying patient information will be released outside of Ambry Genetics as a result of this study. Neither you nor your patient or patient's family will be re-contacted in regards to this study.

This is an optional survey consisting of a 19 item checklist with the option to provide additional information, and is expected to take less than 10 minutes. We do not anticipate that taking this survey will impose any significant risk or inconvenience to you. Participation in this study will not likely provide any direct benefits to you or your patient, however our goal is to share the information obtained in this study so that the findings may benefit the care of future patients and families. This study has been IRB approved. If you have any questions regarding IRB approval for this study, please contact Solutions IRB directly at phone number 1-855-226-4472. If you have any questions or comments about the study, please contact Christina Alamillo, MS, CGC at calamillo@ambrygen.com.

In what ways did the results of exome sequencing affect the management plan for your patient? (check all that apply)

**MEDICATIONS**	**Primary Result**	**Secondary Result**
Prescription of new medication or supplement	☐	☐
Discontinuation of unnecessary medication or supplement	☐	☐
Discontinuation of medication with potential adverse effects	☐	☐
**TESTING AND REFERRALS**		
Referral to additional specialist(s)	☐	☐
Discontinuation of further diagnostic studies	☐	☐
Discontinuation of additional genetic testing	☐	☐
**OTHER MEDICAL MANAGEMENT**		
Investigation for additional manifestations of the genetic condition	☐	☐
Availability of enhanced surveillance and/or prophylactic surgery	☐	☐
Change of prognosis expectations	☐	☐
Availability/eligibility of clinical trial	☐	☐
**PSYCHOSOCIAL**		
Referral to support group or organization	☐	☐
Availability of additional educational services, social services and/or patient advocacy	☐	☐
Altered the family's financial planning	☐	☐
**FAMILY PLANNING**		
Changed presumed inheritance pattern	☐	☐
Established accurate recurrence risks	☐	☐
Enabled reproductive planning/testing options (e.g. preimplantation genetic diagnosis, prenatal diagnosis, and/or decision to have additional children)	☐	☐
Gained option for carrier testing for family members	☐	☐
**OTHER**		
Enabled earlier diagnosis of an affected or pre-symptomatic relative	☐	☐
No significant changes overall	☐	☐

Additional change(s), please specify:
Other comments:

Supplementary References

1. Scocchia, A. *et al.* Clinical whole genome sequencing as a first-tier test at a resource-limited dysmorphology clinic in Mexico. *NPJ Genom Med* **4**, 5, doi:10.1038/s41525-018-0076-1 (2019).
2. Kingsmore, S. F. *et al.* A Randomized, Controlled Trial of the Analytic and Diagnostic Performance of Singleton and Trio, Rapid Genome and Exome Sequencing in Ill Infants. *Am J Hum Genet* **105**, 719-733, doi:10.1016/j.ajhg.2019.08.009 (2019).
3. Hayeems, R. Z. *et al.* The development of the Clinician-reported Genetic testing Utility InDEx (C-GUIDE): a novel strategy for measuring the clinical utility of genetic testing. *Genet Med*, doi:10.1038/s41436-019-0620-0 (2019).
4. PhenoTips. *PhenoTips Care Pathways v.1.2* (2020). Toronto, Canada.
5. Niguidula, N. *et al.* Clinical whole-exome sequencing results impact medical management. *Molecular genetics & genomic medicine* **6**, 1068-1078, doi:10.1002/mgg3.484 (2018).

SUPPLEMENTARY INFORMATION

Medical Genome Initiative Members

Robin Z. Hayeems[1,*], David Dimmock[2], David Bick[3], John W. Belmont[4], Robert C. Green[5], Brendan Lanpher[6], Vaidehi Jobanputra[7], Roberto Mendoza[8], Shashi Kulkarni[9], Megan E. Grove[10], Stacie L. Taylor[4], Euan Ashley[10], Christian R. Marshall[1], Hutton M. Kearney[6]
